# The neuropathological landscape of small vessel disease and Lewy pathology in a cohort of Hispanic and non-Hispanic White decedents with Alzheimer disease

**DOI:** 10.1186/s40478-024-01773-4

**Published:** 2024-05-24

**Authors:** Hsin-Pei Wang, Rebeca Scalco, Naomi Saito, Laurel Beckett, My-Le Nguyen, Emily Z. Huie, Lawrence S. Honig, Charles DeCarli, Robert A. Rissman, Andrew F. Teich, Dan M. Mungas, Lee-Way Jin, Brittany N. Dugger

**Affiliations:** 1 Department of Pathology and Laboratory Medicine, University of California Davis School of Medicine, Sacramento, CA USA; 2 Division of Biostatistics, Department of Public Health Sciences, University of California Davis, Davis, CA USA; 3 Alzheimer’s Disease Research Center, Department of Neurology, University of California Davis School of Medicine, Sacramento, CA USA; 4 Department of Neurosciences, University of California San Diego, San Diego, La Jolla, CA USA; 5 Taub Institute for Research on Alzheimer’s Disease and Aging Brain, Department of Neurology, Department of Pathology and Cell Biology, Columbia University Medical Center, New York, USA; 6 Taub Institute for Research on Alzheimer’s Disease and Aging Brain, Department of Neurology, Columbia University Medical Center, New York, USA

**Keywords:** Autopsy, Neurodegeneration, Vascular pathology, Lewy pathology, Latino, LatinX, Disparities, Dementia, alzheimer disease research centers, Histology

## Abstract

Cerebrovascular and α-synuclein pathologies are frequently observed alongside Alzheimer disease (AD). The heterogeneity of AD necessitates comprehensive approaches to postmortem studies, including the representation of historically underrepresented ethnic groups. In this cohort study, we evaluated small vessel disease pathologies and α-synuclein deposits among Hispanic decedents (HD, n = 92) and non-Hispanic White decedents (NHWD, n = 184) from three Alzheimer’s Disease Research Centers: Columbia University, University of California San Diego, and University of California Davis. The study included cases with a pathological diagnosis of Intermediate/High AD based on the National Institute on Aging– Alzheimer’s Association (NIA-AA) and/or NIA-Reagan criteria. A 2:1 random comparison sample of NHWD was frequency-balanced and matched with HD by age and sex. An expert blinded to demographics and center origin evaluated arteriolosclerosis, cerebral amyloid angiopathy (CAA), and Lewy bodies/Lewy neurites (LBs/LNs) with a semi-quantitative approach using established criteria. There were many similarities and a few differences among groups. HD showed more severe Vonsattel grading of CAA in the cerebellum (p = 0.04), higher CAA density in the posterior hippocampus and cerebellum (ps = 0.01), and increased LBs/LNs density in the frontal (p = 0.01) and temporal cortices (p = 0.03), as determined by Wilcoxon’s test. Ordinal logistic regression adjusting for age, sex, and center confirmed these findings except for LBs/LNs in the temporal cortex. Results indicate HD with AD exhibit greater CAA and α-synuclein burdens in select neuroanatomic regions when compared to age- and sex-matched NHWD with AD. These findings aid in the generalizability of concurrent arteriolosclerosis, CAA, and LBs/LNs topography and severity within the setting of pathologically confirmed AD, particularly in persons of Hispanic descent, showing many similarities and a few differences to those of NHW descent and providing insights into precision medicine approaches.

**Supplementary Information:**

The online version contains supplementary material available at 10.1186/s40478-024-01773-4.

## Introduction

Mixed pathologies are commonly found in the brains of persons diagnosed with dementia. The terminology of “mixed pathologies” refers to the co-occurrence of multiple pathological findings within the same individual [1, 2]. The three most common mixed pathologies within the dementia spectrum are Alzheimer disease (AD), cerebrovascular disease (CVD), and Lewy body disease (LBD) [2, 3, 4]. Cohort studies investigating individuals with pathological diagnoses of AD reported a high rate of concomitant vascular and Lewy body (LB) pathologies [2, 5–7]. The presence of these combined pathological features can complicate diagnosis, prognosis, and treatment strategies. Concomitant pathologies can also present substantial hurdles in identifying biomarkers and accurate assessment of disease progression timelines [1, 2]. Given the multifactorial nature of dementia as well as the increasing demographic diversity of the United States, comprehensive approaches examining diverse cohorts are essential to unravel the intricate contributions of these diseases, their potential interactions, and the complex conditions observed [8].

Cerebrovascular disease (CVD) is very heterogeneous and can encompass multiple pathologies such as infarcts, atherosclerosis, arteriolosclerosis, and cerebral amyloid angiopathy (CAA) [2, 9]. Among the most common vascular pathologies, arteriolosclerosis and CAA, which we will collectively refer to as small vessel disease (SVD) for this paper, have been increasingly recognized as key contributors to cognitive impairment and increased risk of dementia [9–14]. Arteriolosclerosis, involving thickening of vessel walls with subsequent narrowing of the lumen, is associated with cardiovascular risk factors, such as hypertension and diabetes [13, 15], and mostly found in the deep white matter of basal ganglia, frontal, temporal, and occipital cortices [9, 16–18]. The deposition of amyloid protein in the vessel walls, defined as CAA, is predominately present in the leptomeninges and grey matter of occipital, temporal, and frontal cortices [19–21].

Lewy body disease (LBD) is a pathological term with a spectrum of clinical syndromes including Parkinson disease, Parkinson disease dementia (PDD), and dementia with Lewy bodies (DLB) and is characterized by the presence of Lewy bodies in the brain [22, 23]. LBD is the second most common neurodegenerative disease following AD, although individuals often exhibit co-existing AD-related pathologies [6, 24]. Similar to AD, LBD is a substantial socioeconomic burden within the United States [25]. This concerns the affected persons, their caregivers, and the healthcare system, with far-reaching negative socioeconomic implications, especially for historically marginalized populations [26–29]. Despite its widespread impact, research investigating LBD progression and its co-occurrence of AD pathology has been predominantly centered on individuals of European ancestry, with few studies examining ethnically diverse cohorts, particularly those of Hispanic descent [8]. This dearth of diversity in research cohorts may limit the generalizability of the current literature, ultimately limiting advancement in diagnostic and therapeutic efforts that are inclusive for all individuals.

At the 2022 Alzheimer’s Disease-Related Dementias (ADRD) Summit, experts addressed the research priorities concerning health equity and multiple etiology dementias [1]. Given the growing diversity within persons afflicted with dementia and a dearth of studies examining persons of Hispanic descent, we sought to include a more extensive representation of Hispanic decedents into the current cohort to approach mixed pathologies comprehensively. Most studies focus on concurrent AD and SVD, or AD pathologies within LBD [30, 31]. Hence, using a cohort of Hispanic and non-Hispanic White decedents with AD in the current study, we examined pathological burdens with established semi-quantitative scoring systems of arteriolosclerosis [9, 15], CAA [32, 33], and Lewy pathologies [22, 23] in select neuroanatomic areas across three Alzheimer’s Disease Research Centers (ADRCs): Columbia University, University of California San Diego (UCSD), and University of California Davis (UCD).

## Materials and methods

### Cohort and area selection

For a detailed description of overall methods and cohort selection, please refer to Scalco et al., 2023 [34]. Briefly, participants were selected based on a pathological diagnosis of Intermediate/High AD [35, 36], as well as self-reported identification of ethnicity as Hispanic and non-Hispanic White descent utilizing data from the National Alzheimer’s Coordinating Center (NACC) uniform data set (UDS) [37]. A 2:1 random sample of non-Hispanic White decedents was chosen, stratified by age group, sex, and center to be comparable to Hispanic decedents. The final sample consisted of 276 individuals (92 Hispanic decedents, 184 non-Hispanic White decedents), including Hispanic decedents from Mexican, Caribbean (Cuban, Puerto Rican, and Dominican), and other origins (Central and South America). Cases without available pathology data and individuals with ethnicities other than Hispanic or non-Hispanic White descent were excluded. We follow the guidelines provided by JAMA regarding the usage of terminology when reporting race and ethnicity [38].

The selection of brain areas for analysis was based on 2012 NIA-AA guidelines availability [36, 39], having similar sampling procedures across all three centers. The temporal and frontal cortices, amygdala, substantia nigra, and locus coeruleus were assessed for α-synuclein deposits in the form of LBs/Lewy neurites (LNs). The temporal, parietal, and frontal lobes, posterior hippocampus, and cerebellum were assessed for CAA. White matter regions of temporal, parietal, and frontal lobes were assessed for arteriolosclerosis. Additional details on scoring systems are located below.

### Clinical comorbidity data

Available information on select clinical comorbidities was documented by retrieved data from the NACC UDS or similar forms employed by each ADRC [37]. Presence of diabetes, hypertension, depression, trans ischemic attack, hyperlipidemia, and stroke was recorded in the UDS as active or inactive throughout the history of diagnosis and/or if the participant was mentioned to be taking medication to treat these conditions.

### Histology and assessments

Methodologies for sample preparation, cutting, as well as amyloid-β and tau immunohistochemistry have been previously described [34]. The deparaffinized slides for α-synuclein assessment underwent a 30-minute steam pretreatment in distilled water using an Oster pressure steamer, followed by a 5-minute incubation in a Proteinase K solution (Sigma-Aldrich, Cat # P8038, St. Louis, MO, USA) prepared with 0.1 g of Proteinase K and 400 milliliters of distilled water. After pretreatment, slides were placed on a DAKO AutostainerLink48 and then subjected to the following: after rinsing in deionized water for 1 min, 0.3% Hydrogen Peroxide was applied to block the endogenous peroxidase for 10 min. Slides were then rinsed with wash buffer (Agilent Technologies, Cat # S3006, Santa Clara, CA, USA) and deionized water each for 1 min. Slides were then incubated for 30 min with a monoclonal antibody LB509 against α-synuclein (1:80 dilution, Invitrogen, Cat # 180215, Waltham, MA, USA). After two 1-minute rinses with wash buffer and deionized water, the primary antibody was labelled by the EnVision + HRP. Mouse (Agilent Technologies, Cat # K400111-2) for 20 min. Another 2 min of rinsing with wash buffer and deionized water was applied before the 10-minute incubation of DAB + as the substrate-chromogen (Agilent Technologies, Cat # K346811-2). Next, slides were rinsed with wash buffer for 5 min and deionized water for 1 min, and then hematoxylin (1:4, American Mastertech, Cat # HXHHEGAL, Lodi, CA, USA) as the counterstain was applied for 5 min. After 2 min of rinsing with wash buffer and deionized water, the visualization of α-synuclein was finished. Standard procedures were adhered to antibody staining using automated machines (DAKO AutostainerLink48, Agilent Technologies), ensuring the inclusion of appropriate positive and negative controls for each specific antibody. The UCD Histology Core conducted all staining and immunohistochemistry procedures, complying with all Federal, State of California, and UCD guidelines and regulations [34].

For assessment of arteriolosclerosis, slides were subjected to Hematoxylin and Eosin (H&E) staining. H&E staining consisted of three xylene rinses (5, 5, and 3 min), followed by four 30-second washes with 100%, 100%, and 95% ethanol, and water. Following an 11-minute incubation in Harris hematoxylin (American Mastertech, Cat # HXHHEGAL), the slides were rinsed for 1 min, differentiated using 1% acid alcohol for 3 s, and then washed for 30 s. Next, four 30-second washes with water, bluing reagent (prepared weekly by 15 g of Lithium carbonate with 4000 milliliters of distilled water), water, and 80% ethanol, preceded a 4.5-minute incubation with eosin Y (American Mastertech, Cat # STE0157). The slides underwent three 45-second rinses with 100% ethanol, followed by clearing in three xylene baths (60, 45, and 45 s).

Stained slides were digitally scanned using the Zeiss Axio Scan Z.1 scanner. H&E slides were scanned at 20 × (0.22 μm/pixel) magnifications and α-synuclein and amyloid-β stained slides at 40 × (0.11 μm/pixel) magnifications to acquire whole slide imaging. The resulting digital images were saved in the czi format with a compression rate of 60%. An expert (BND), blinded to the demographic, pathological, clinical, and genetic data on the cases as well as ADRC origin, performed semi-quantitative histopathological assessments of each area and stain. The assessments included the evaluation of CAA, arteriolosclerosis, and LBs/LNs pathologies, and followed the guidelines outlined in the NACC Neuropathology form version 10, Vonsattel et al., and dementia with Lewy bodies consortium [9, 15, 22, 23, 32, 33]. The detailed scoring systems of amyloid plaques and tau pathologies has been previously published elsewhere [34].

The evaluation of CAA utilized amyloid-β-stained slides from the cerebellum, posterior hippocampus, and frontal, parietal, and temporal lobes by assessing the density of positive vessels and the severity of individual vessels within each area, using a modified NACC and Vonsattel’s scoring system [32, 33]. The modifications refer to the use of amyloid-β staining as well as adapting the global scale to the specific tissue section. On a specific region of CAA, the expert assigned a grade (0 = none - absent, 1 = mild - scattered positivity in parenchymal and/or leptomeningeal vessel in focal areas within the tissue sample, 2 = moderate - intense positivity in many parenchymal and/or leptomeningeal vessels within the tissue sample, 3 = severe - widespread (throughout the tissue sample) intense vessel positivity). The CAA Vonsattel grade (ranging from grade 0 to 4) was determined by considering the majority of the severity level within the tissue observed [33]. For a Vonsattel grade 1 - most affected vessels contained amyloid-β deposits in otherwise normal leptomeninges in an incomplete rim around muscle fibers; Vonsattel 2 - most affected vessels had the media of vessels fully replaced by amyloid-β and walls were thickened; Vonsattel 3 - most affected vessels had total amyloid-β replacement of media and cracking of vessel walls creating a vessel within vessel appearance; Vonsattel 4 - majority of affected vessels showed scarring and necrosis with traces of intermingled amyloid-β deposits. It should be emphasized a score was assigned only when the majority of vessels in a region exhibited that level of severity, rather than scoring solely based on the presence of a single vessel with that severity. We utilized previously published scales to provide a semi-quantitative assessment of arteriolosclerosis based on the appearance of the majority of vessels (0 = none - normal, 1 = mild - mild thickening of vessel medial, mild fibrosis, 2 = moderate - partial loss of smooth muscle cells in the media, moderate hyaline fibrosis, 3 = severe - complete loss of smooth muscle cells in media, severe hyaline fibrosis, lumen stenosis) in the white matter of the frontal, parietal, and temporal lobes [9, 15]. Assessment of α-synuclein pathologies, LBs and LNs, utilized the four stages as defined by the dementia with Lewy bodies consortium (0 = absent, 1 = mild - sparse LBs or LNs, 2 = moderate - more than one LB per high power field and sparse LNs, 3 = severe - more than four LBs and scattered LNs in low power field, 4 = very severe - numerous LBs and LNs) [22, 23].

### Statistical analysis

Demographic and clinical characteristics of the cohort were summarized descriptively and compared for Hispanic and non-Hispanic White decedents, using means, standard deviations (SD), and t-tests for continuous variables and percentage and chi-square tests for categorical variables. Neuropathological findings were all reported as ordinal scales reflecting density in chosen regions; they were summarized descriptively by median, minimum, and maximum values, and compared between groups by Wilcoxon two-sample tests with ties correction. Ordinal logistic regression models were then used to compare the level of neuropathology findings after adjusting for age, sex, and center. The Hispanic decedents were further categorized by self-reported ethnicity (Caribbean, Mexican, and Others), and differences across ethnic Hispanic groups further compared via ordinal logistic regression adjusted for age and sex and corrected for multiple comparisons by false discovery rate. All analyses were carried out in SAS version 9.4. Figures were created using Biorender (Biorender.com) and R Studio package ggplot2 [40].

## Results

### Demographic and clinical characteristics

In the cohort, persons of Hispanic descent were comparable to persons of non-Hispanic White descent in age at death (means 81 ± 9 and 82 ± 9 years) and sex ratios (59% and 60% female), as well as apolipoprotein E (APOE) ε4 carrier status (55% and 57%) (Table 1). Hispanic decedents had fewer years of formal education, with a mean of 9.7 years compared to 14.6 years for non-Hispanic White decedents. Examining data from NACC, within the cohort, persons of races other than White included Hispanic decedents who identified as African American (n = 4, all with Caribbean origins), other (n = 27, of these 27 with respect to ethnicity: 18 with Caribbean origins, 1 with Mexican origins, and 8 with other origins), and unknown (n = 16, 2 with Caribbean origins, 2 with Mexican origins, and 12 with other origins). The proportions with a pathological diagnosis of AD only (AD lacking a concomitant diagnosis of CVD and/or LBD) were identical (37%), and with mixed AD/CVD as well were very similar (35% and 38%), but Hispanic decedents had greater frequencies of LBD (29% compared to 19%). Certain clinical comorbidities were more common among Hispanic decedents: diabetes (24% vs. 8%), hypertension (67% vs. 52%), and stroke (24% vs. 14%), while high cholesterol was more common in non-Hispanic Whites decedents (58% vs. 39%).

**Table 1:** Demographics, select clinical comorbidities, and presence of regional pathology divided by ethnic group (n = 276)

	NHWD (n = 184)	HD (n = 92)	P value
**Demographics**			
Age at death (years), mean (SD)	82.2 (8.7)	81.4 (9.2)	0.47*
Education (years), mean (SD) total	14.6 (3.0) 181	9.7 (4.6) 86	<0.01*
Sex (female), N (%)	110 (60.0%)	54 (58.7%)	0.86§
APOE ε4 (at least one), N (%) total	75 (56.8%) 132	41 (54.7%) 75	0.76§
**Contributing Pathology**			
AD only, N (%)	68 (37.0%)	34 (37.0%)	1§
Mixed AD/LBD, N (%)	35 (19.0%)	27 (29.3%)	0.05§
Mixed AD/CVD, N (%)	69 (37.5%)	32 (34.8%)	0.66§
**Clinical Comorbidities**			
Diabetes, N (%) total	13 (8.4%) 154	18 (24.0%) 75	<0.01§
Hypertension, N (%) total	81 (52.3%) 155	50 (66.7%) 75	0.04§
High Cholesterol, N (%) total	77 (57.5%) 134	24 (38.7%) 62	0.01§
Stroke, N (%) total	25 (14.2%) 176	21 (23.6%) 89	0.06§
Trans ischemic attack, N (%) total	16 (12.0%) 133	8 (15.4%) 52	0.54§
Depression, N (%) total	35 (23.8%) 147	16 (22.5%) 71	0.84§
**Arteriolosclerosis Presence**			
Temporal lobe, N (%) total	162 (99.4%) 163	77 (97.5%) 79	0.25†
Parietal lobe, N (%) total	170 (96.0%) 177	78 (89.7%) 87	0.05†
Frontal lobe, N (%) total	165 (97.1%) 170	80 (94.1%) 85	0.31†
**CAA Presence**			
Temporal lobe, N (%) total	124 (72.5%) 171	62 (75.6%) 82	0.60§
Parietal lobe, N (%) total	111 (68.1%) 163	59 (72.0%) 82	0.54§
Posterior Hippocampus, N (%) total	89 (53.6%) 166	55 (66.3%) 83	0.06§
Frontal lobe, N (%) total	126 (75.9%) 166	67 (78.8%) 85	0.60§
Cerebellum, N (%) total	128 (70.7%) 181	70 (83.3%) 84	0.03§
**LBs/LNs Presence**			
Temporal cortex, N (%) total	45 (25.3%) 178	30 (36.6%) 82	0.06§
Amygdala, N (%) total	64 (38.8%) 165	35 (44.3%) 79	0.41§
Substantia nigra, N (%) total	53 (29.4%) 180	31 (38.3%) 81	0.16§
Frontal cortex, N (%) total	25 (14.5%) 172	22 (26.2%) 84	0.02§
Locus coeruleus, N (%) total	45 (27.3%) 165	26 (32.9%) 79	0.36§

Total represents the number of cases with available data on the specific variable. All cases had available data on age at death, sex, and primary/secondary pathology diagnoses. Items presented are from historical data collected by each center albeit regional pathology scores

APOE ε4, apolipoprotein E ε4; NHWD, non-Hispanic White decedents; HD, Hispanic decedents; LBD, Lewy body disease; CVD, cerebrovascular disease; CAA, cerebral amyloid angiopathy; LBs/LNs, Lewy bodies/Lewy neurites

§Chi-square test

†Fisher exact test

*T-test

### Neuropathology

**Fig. 1:**
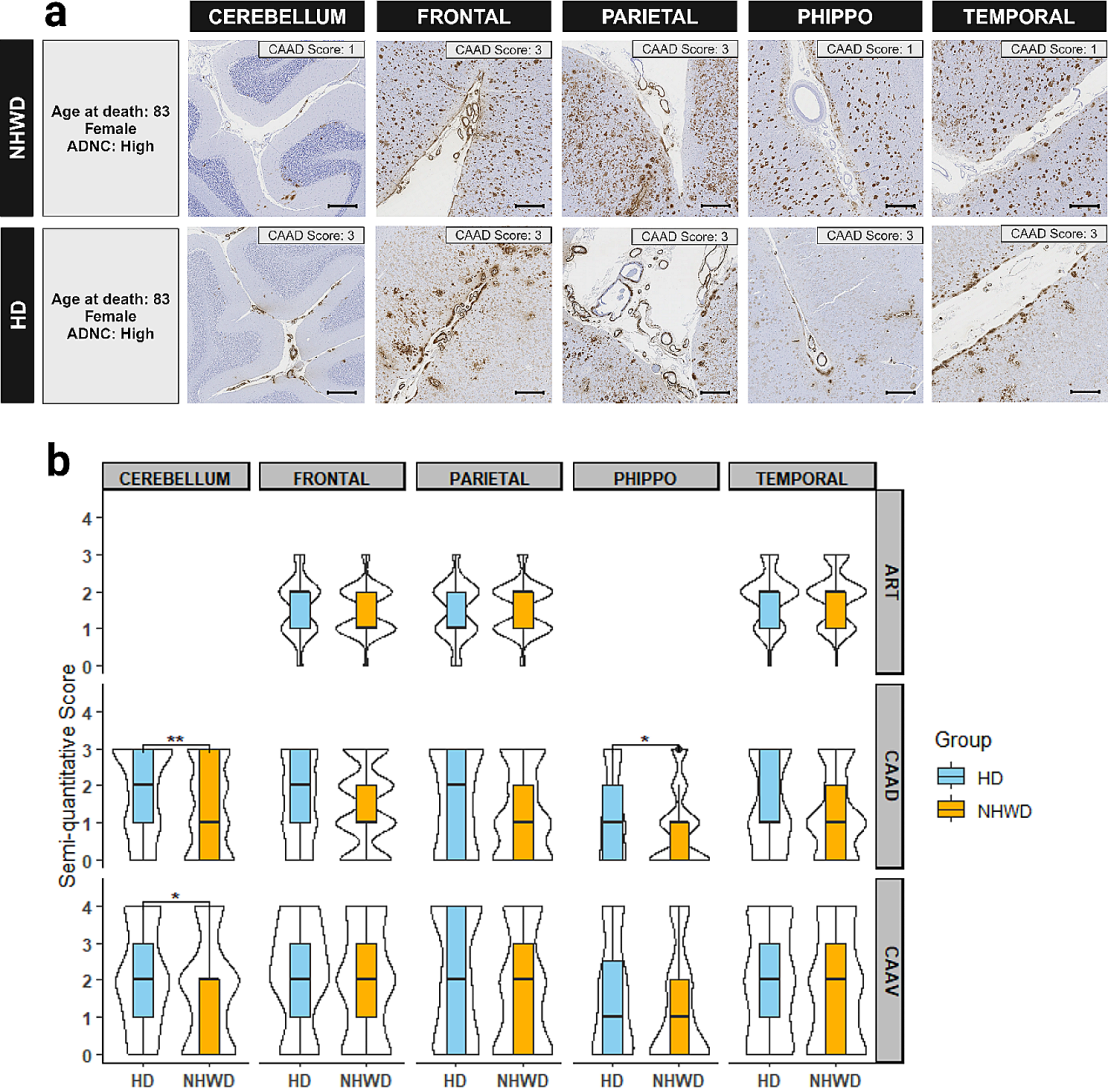
Severity of cerebral amyloid angiopathy (CAA) and arteriolosclerosis (ART) in select neuroanatomic regions. **a** Examples of cerebral amyloid angiopathy density (CAAD) in frontal, parietal, and temporal lobes, posterior hippocampus (PHIPPO), and cerebellum; overall regional density scores are listed in panel within each image. Cases were selected based on heritage group and center, having similar age at death, sex, and AD likelihood (ADNC = Alzheimer’s disease neuropathologic changes). Scale bar = 500 μm. **b** Violin plots of the severity of ART, CAAD, and cerebral amyloid angiopathy Vonsattel score (CAAV) in select brain areas between Hispanic and non-Hispanic White decedents. CAAD and CAAV were assessed in five areas: frontal, parietal, and temporal lobes, posterior hippocampus (figure represents collateral sulcus), and cerebellum. ART was assessed in the frontal, parietal, and temporal white matter. The violin plots indicated the distribution of the data while the boxes showed the first and third quartiles, median, and range

**Fig. 2:**
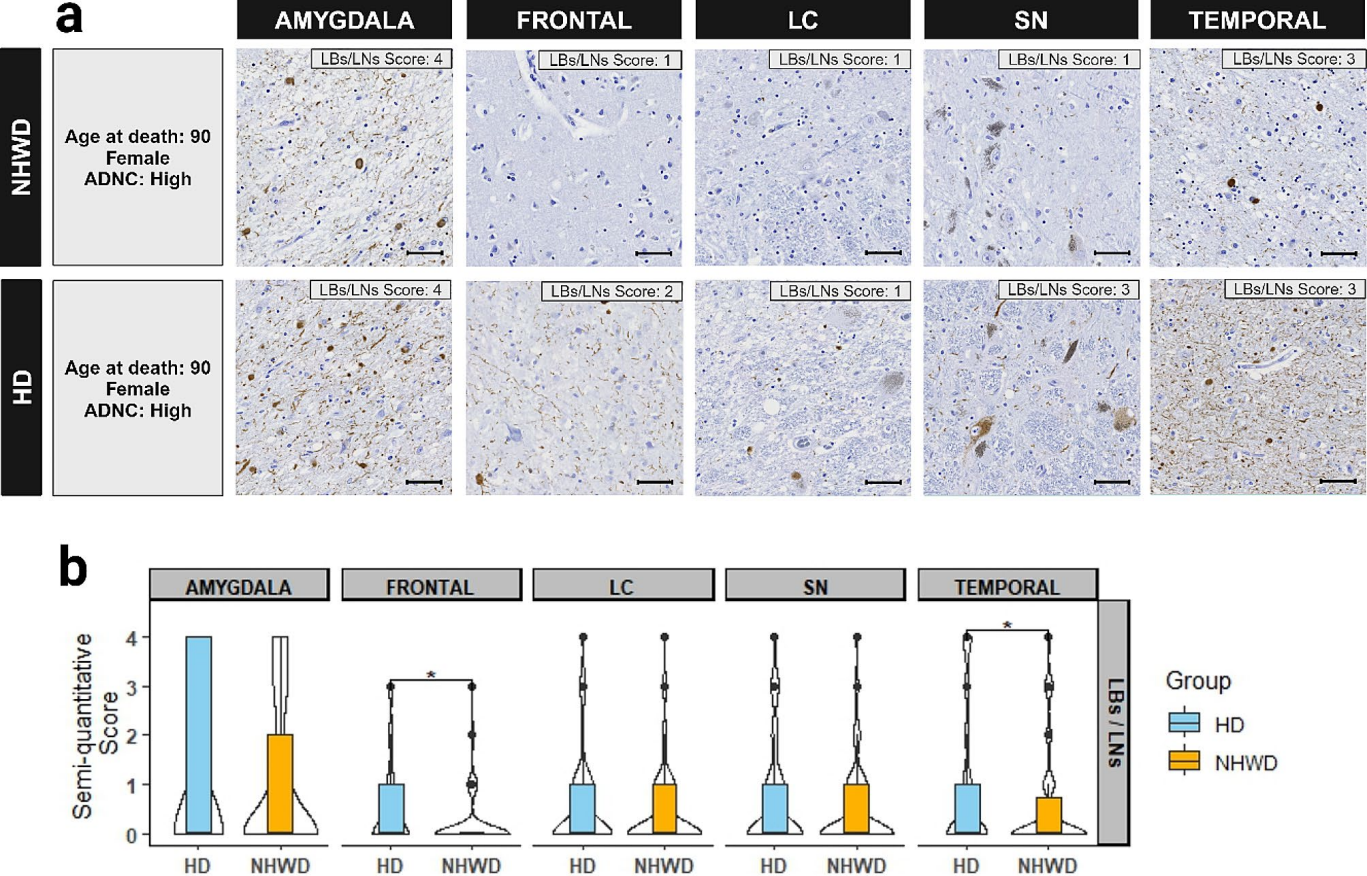
α-synuclein deposit densities of Lewy bodies/Lewy neurites (LBs/LNs) in select neuroanatomic regions. **a** Examples of α-synuclein staining densities in frontal and temporal cortices, amygdala, substantia nigra (SN), and locus coeruleus (LC); overall regional density scores are listed in panel within each image. Cases were selected based on heritage group and center, having similar age at death, sex, and AD likelihood (ADNC = Alzheimer’s disease neuropathologic changes). Scale bar = 50 μm. **b** Violin plots of the distribution of LB/LN density scores in five selected brain areas (frontal and temporal cortices, amygdala, SN, and LC) between Hispanic decedents (HD) and non-Hispanic White decedents (NHWD). The violin plots indicated the distribution of the data while the boxes showed the first and third quartiles, median, and range

All neuropathology measures followed an ordinal scale; while medians were generally similar and often low, the distribution above the 50th percentile, representing a subgroup with more severe pathology, sometimes differed between groups, as detected by the Wilcoxon test. Arteriolosclerosis was less frequent in the parietal lobe in Hispanic compared to non-Hispanic White decedents (Table 1) while similar in both groups across three lobes with semi-quantitative analysis (Table 2). Cerebral amyloid angiopathy (CAA) was similar in temporal, parietal, and frontal lobes, but greater in Hispanic decedents in the cerebellum in both density and Vonsattel grade (Tables 1 and 2), as well as in density in posterior hippocampus (Fig. 1). The median LBs/LNs score detecting α-synuclein was 0 for all five regions for both Hispanic and non-Hispanic White decedents (Table 2), indicating half or more cases did not have LBs/LNs pathology. But among the remainder, increasing density of LBs/LNs was denoted in Hispanic than non-Hispanic White decedents in both the temporal and frontal cortical regions (Fig. 2). Results were similar in ordinal logistic regressions adjusted for age and sex.

**Table 2:** Severity scores of cerebrovascular and Lewy-related neuropathologies in select brain areas by ethnic group (n = 276)

	NHWD (n = 184)	HD (n = 92)	P value (Wilcoxon two-sample test)
**Arteriolosclerosis** (0–3: 0 = None, 1 = Mild, 2 = Moderate, 3 = Severe) [9, 15]			
Temporal lobe, median (min, q3, max)	2 (0,2,3)	2 (0,2,3)	0.60
total	163	79	
Parietal lobe, median (min, q3, max) total	2 (0,2,3) 177	1 (0,2,3) 87	0.39
Frontal lobe, median (min, q3, max) total	1 (0,2,3) 170	2 (0,2,3) 85	0.27
**CAA Density** (0–3: 0 = None, 1 = Mild, 2 = Moderate, 3 = Severe) [32]			
Temporal lobe, median (min, q3, max)	1 (0,2,3)	1 (0,3,3)	0.17
total	171	82	
Parietal lobe, median (min, q3, max) total	1 (0,2,3) 164	2 (0,3,3) 82	0.11
Posterior Hippocampus, median (min, q3, max)	1 (0,1,3)	1 (0,2,3)	0.01
total	166	83	
Frontal lobe, median (min, q3, max) total	1 (0,2,3) 165	2 (0,3,3) 85	0.28
Cerebellum, median (min, q3, max) total	1 (0,3,3) 181	2 (0,3,3) 84	0.01
**CAA Vonsattel** (0–4: Grade 0 ~ 4) [33]			
Temporal lobe, median (min, q3, max)	2 (0,3,4)	2 (0,3,4)	0.35
total	171	82	
Parietal lobe, median (min, q3, max) total	2 (0,3,4) 163	2 (0,4,4) 82	0.14
Posterior Hippocampus, median (min, q3, max)	1 (0,2,4)	1 (0,3,4)	0.08
total	166	83	
Frontal lobe, median (min, q3, max) total	2 (0,3,4) 166	2 (0,3,4) 85	0.32
Cerebellum, median (min, q3, max) total	2 (0,2,4) 181	2 (0,3,4) 84	0.04
**LBs/LNs** (0–4: 0 = Absent, 1 = Mild, 2 = Moderate, 3 = Severe, 4 = Very Severe) [22, 23]			
Temporal cortex, median (min, q3, max)	0 (0,1,4)	0 (0,1,4)	0.03
total	178	82	
Amygdala, median (min, q3, max)	0 (0,2,4)	0 (0,4,4)	0.11
total	165	79	
Substantia nigra, median (min, q3, max)	0 (0,1,4)	0 (0,1,4)	0.09
total	180	81	
Frontal cortex, median (min, q3, max)	0 (0,0,3)	0 (0,1,3)	0.01
total	172	84	
Locus coeruleus, median (min, q3, max)	0 (0,1,4)	0 (0,1,4)	0.33
total	165	79	

Total represents the number of cases with available data on the specific variable

NHWD, non-Hispanic White decedents; HD, Hispanic decedents; CAA, cerebral amyloid angiopathy; LBs/LNs, Lewy bodies/Lewy neurites

The Hispanic decedents came from three centers, with different ethnic mixes; the largest groups had Mexican ethnic origins and Caribbean origins, with Others representing various countries in Central and South America, combined for purposes of analysis. UC Davis contributed 7 with Mexican origins and 10 Others, UC San Diego 23 with Mexican origins and 8 Others, and Columbia 1 with Mexican origins, 7 Others, and 36 with Caribbean origins. We compared neuropathology across the three Hispanic ethnic subgroups of Mexican, Caribbean, and Others, with ordinal logistic regression adjusted for age and sex but not center as it was confounded with ethnicity. The primary finding was LBs/LNs pathology was elevated in both temporal and frontal cortices among the Caribbean decedents compared both to non-Hispanic White and other Hispanic ethnic groups (Table 3). There were no differences found across Hispanic ethnicities in arteriolosclerosis or CAA presence or densities.

**Table 3:** Regression model analysis of Lewy pathology. Caribbean decedents have significantly higher odds of having denser Lewy pathology than NHWD and Mexican decedents in the temporal and frontal cortices

	**Temporal Lewy Pathology**	Frontal Lewy Pathology
	OR (95% CI)	OR (95% CI)
**Ethnicity**		
Mexican vs. NHWD	0.37 (0.1, 1.3)	0.39 (0.08, 1.76)
Caribbean vs. NHWD	4.42 (2.11, 9.25)*	6.58 (2.85, 15.22)*
Others vs. NHWD	1.43 (0.51, 4.0)	1.58 (0.47, 5.28)
Mexican vs. Others	0.26 (0.05, 1.22)	0.24 (0.04, 1.55)
Caribbean vs. Others	3.1 (0.95, 10.07)	4.17 (1.09, 16.04)
Caribbean vs. Mexican	12.05 (3.0, 48.27)*	17.06 (3.32, 87.66)*
**Sex**		
Female vs. Male	1.42 (0.78, 2.58)	1.04 (0.5, 2.15)
**Age of death**		
Age (year)	0.98 (0.95, 1.02)	1.00 (0.96, 1.05)

*Indicates P-values remain significant (< 0.05) after false discovery rate

NHWD, non-Hispanic White decedents

## Discussion

We examined regional burdens of arteriolosclerosis, CAA, and LBs/LNs pathologies through a semi-quantitative assessment within a research-based autopsy-confirmed AD cohort, comprised of Hispanic and non-Hispanic White decedents. Our findings confirm previous cohort studies denoting high frequencies of mixed pathologies in individuals with advanced AD pathology. Arteriolosclerosis was present in over 90%, CAA in 54-83%, and LBs/LNs in 15-39% across neuroanatomic regions in this cohort. There were many similarities across groups with some differences. Compared with non-Hispanic White decedents, Hispanic decedents displayed greater frequencies of CAA in the cerebellum (83% vs. 71%), increased CAA density in the posterior hippocampus and cerebellum, and higher frequency of LBs/LNs in the frontal cortices (27% vs. 15%) and temporal cortices (37% vs. 25%). This study provides a deeper phenotype denoting many similarities and some differences across ethnicity in persons with pathological AD, which may aid with precision medicine approaches for AD and related disorders.

There have been few studies to our knowledge, examining LBD with respect to Hispanic ethnicity [8, 41–43]. In a cohort assessed from the NACC dataset, which included participants with neuropathologically confirmed transitional (limbic) or diffuse (neocortical) LBD, Hispanic decedents (n = 54) were reported to have more transitional LBD compared with non-Hispanic White decedents (n = 141) [42]. Previous works in individuals with dementia, regardless of underlying pathology, who had autopsy at the UCD ADRC, reported a higher frequency of mixed LBD and AD pathology in persons of Hispanic descent (25%) when compared to persons of non-Hispanic White descent (18%) [41]. The current study included cases from UCD and two additional centers (Columbia and UCSD) with a focus on AD. In this study of three centers, higher frequencies of AD with LBD were found in Hispanic (29%) compared to non-Hispanic White decedents (19%). We also found an increased density of LBs/LNs deposits in the temporal and frontal cortical regions in Hispanic decedents, providing additional anatomic specificity. In contrast to our current work, another study of 1625 participants with pathological AD diagnoses, including 67 Hispanic decedents, showed no significant differences in LBD frequencies between Hispanic (25%) and non-Hispanic White decedents (24%) [43]. Despite the similarity in the design of these two studies, the observed variation could be explained by their sample size, most of which were of Caribbean origin (n = 46). Our study did have similar overall frequencies of LB types within the setting of AD; although prior studies did not examine ethnicity. In a cohort of 522 participants aged 50 or older, neocortical and amygdala predominant LBD were frequently observed in people with Intermediate (40%) and High (62%) AD pathology [31]. In our study extrapolating data from Table 1, we had 32% of cases with neocortical (n = 76) or amygdala (n = 10) predominant LBD. More research is needed to confirm whether concomitant Lewy related pathologies in AD could relate to increased cognitive decline, age of onset, or progression of AD, particularly in persons of Hispanic descent.

A recent review compiling neuropathological studies on persons of Hispanic descent, revealed multiple findings without a unifying agreement regarding AD and CVD [8]. These various results indicate the heterogeneity within persons of Hispanic descent and emphasize the concerns of the sample size of Hispanic cohorts in previous studies. Comprehensive clarification of the variations across studies involving persons who identified as Hispanic will not be elucidated until greater diversity is included in AD research. There was no statistically significant difference in the frequency of mixed AD with CVD between Hispanic (35%) and non-Hispanic White decedents (38%) in the present study. Regarding select SVD pathologies and clinical comorbidities, differences were observed in CAA density, diabetes, hypertension, and high cholesterol, but not in arteriolosclerosis. In a previous investigation, examining individuals with dementia (n = 423), Hispanic decedents (n = 28) had higher frequencies of CVD (21% vs. 4%) and mixed AD with CVD (54% vs. 28%), as well as higher rates of severe arteriolosclerosis (21% vs. 7%) and CAA (11% vs. 5%), in comparison to non-Hispanic White decedents (n = 360) [41]. This positive association between arteriolosclerosis and Hispanic decedents was not consistent with the current results perhaps due to differences in inclusion/exclusion criteria. With respect to CAA, prior works have demonstrated within neuropathologically confirmed AD (n = 425) participants with severe CAA (n = 193) were more likely to be Hispanic (7%) rather than non-Hispanic White decedents (1%) [44]. Similarly, in the UCSD ADRC cohort of persons with autopsy-confirmed AD, it was observed Hispanic decedents (n = 14) had more frequent moderate/severe CAA than non-Hispanic White decedents (n = 20) [45]. These results support more frequent presence of severe CAA among persons of Hispanic descent of which is consistent with our results. With our study, we reveal increased CAA densities in the cerebellum, providing more insight into potential regional neuroanatomic disease mechanisms in the setting of AD.

Although we employed carefully designed methodologies, limitations were present in our cohort study. Caution is advised when extrapolating these results to a broader population since they may not be fully generalizable. Further research having more inclusive cohorts across disease spectrums, including population- or community-based cohorts, is warranted. With all studies on decedents, cohorts are highly selective as they are based on persons who consent for brain donation [48]. Differences and similarities compared to other studies may be due to cohort inclusion/exclusion criteria as well as how data were assessed. For the UCSD ADRC, persons were excluded from participation if they had insulin dependent diabetes, and/or major stroke [34, 46], which could result in exclusion of persons with severe CVD. In addition, our cohort had an inclusion criterion of only participants with Intermediate/High AD pathology. These criteria may have excluded the spectrum of other dementia subtypes such as LBD and CVD, which can have different frequencies among individuals. Regarding the CAA assessment, we analyzed scores per region without distinguishing capillary, cortical, or leptomeningeal CAA separately. Evaluation of CAA as well as the other pathologies did not involve specific subregions but throughout the entire sampled region; for example, the posterior hippocampus was examined as an intact region- of which included anatomic regions of the subiculum, entorhinal cortex, parahippocampal gyrus, collateral sulcus, and fusiform gyrus. A recent study with pure or mixed AD with advanced amyloid-β pathology (n = 73), not denoting ethnicity, showed more leptomeningeal CAA (44%) than parenchymal CAA (19%) [47]. Future studies could subcategorize CAA into capillary, cortical, and/or leptomeningeal as these subcategories could have independent associations with neuropathologies and/or demographic variables. This can also be the case with LBs/LNs, as we examined them together. Lastly, as these are archival samples, and certain processes were done before this study, there were certain variables we were unable to account for. There was evidence of over-fixation, distinguished by the appearance of formalin crystal artifact (Supplementary Fig. 1), present in some anatomic regions in about a third of cases. Over-fixation may affect immunohistochemical stains, leading to potential false negatives, especially with respect to LNs as these pathologies cannot be appreciated upon H&E.

Despite the limitations, there are several strengths of our study. First, it is the most comprehensive autopsy study of persons of Hispanic descent examining SVD and LBs/LN pathology in the setting of pathological AD evaluating multiple neuroanatomic areas. Our inclusion criteria and analytical methodology ensured a considerable degree of homogeneity in terms of age, sex, APOE ε4 allele frequency, and final pathological diagnoses in our study cohort (Table 1). We focused on participants with pathological AD diagnoses of Intermediate/High, aiming to determine frequencies and densities of SVD and LBs/LNs pathologies based on ethnicity under the severe burdens of AD pathologies. Second, we examined and analyzed semi-quantitative scores to evaluate regional burdens of SVD and LBs/LNs in addition to presence/absence. Dichotomous analysis of pathology can have selected advantages, such as getting greater consensus in cases of low inter-rater reliability when assessing different severity of white matter arteriolosclerosis [9]. However, analyzing presence/absence or collapsing semi-quantitative scores into dichotomous categories could oversimplify the data and limit comprehensive interpretation of the findings, yielding different statistical significance as the inconsistent significance of SVD pathologies in the current study (Tables 1 and 2). Many times, pathology is presented as a global score without providing topographical distribution or pathological severity of concomitant SVD and α-synuclein burdens in the setting of AD. Our study fills the gap and reveals similarities and distinct neuroanatomic patterns of pathology in Hispanic and non-Hispanic White decedents. These investigations highlight the importance of the analysis method and delve into deeper phenotypes of SVD and LBs/LNs between Hispanic and non-Hispanic White decedents, providing potential insights into similarities as well as distinct AD progression in different ethnoracial groups.

In conclusion, results of this cohort study demonstrate, after controlling for age, sex, and center of origin, Hispanic decedents with Intermediate/High AD pathology have many similarities to non-Hispanic White decedents with Intermediate/High AD pathology. There were some differences noted; Hispanic decedents within the cohort exhibit significantly greater CAA and LBs/LNs burdens in select regions compared with non-Hispanic White decedents. Our current and previous work consistently present how ethnicity may relate to neuropathological differences in AD hallmarks, SVD, and LBs/LNs, emphasizing the demand for in-depth phenotyping in AD among a diverse cohort due to the comorbidity of the disease. Further research should involve more diverse cohorts, including subgroups within non-Hispanic White, as well as additional underrepresented groups and subgroups such as persons who identify as Asian, African American, and Native American to achieve more generalizable results. In addition, researchers should consider including other common CVD into analysis, such as infarcts, hemorrhages, mineralized blood vessels, and white matter rarefaction, given the heterogeneous nature of AD. It is also important to consider clinical comorbidities within the current cohort, including diabetes, hypertension, and high cholesterol, which we plan to examine in future studies. These efforts will play a crucial role in the inclusion of historically marginalized groups, particularly persons who identify as Hispanic. With a diverse cohort in dementia studies, the findings from various studies can be more generalizable for the development of precision medicine.

### Electronic supplementary material

Below is the link to the electronic supplementary material.

Supplementary Material 1 Supplementary Material 1

## Data Availability

The datasets that support the findings of this study are not publicly available due to privacy or ethical restrictions but are available on reasonable request from the corresponding author.

## Abbreviations

AD: Alzheimer disease
ADRCs: Alzheimer’s Disease Research Centers
CAA: cerebral amyloid angiopathy
CVD: cerebrovascular disease
DLB: dementia with Lewy bodies
HD: Hispanic decedents
LBs: Lewy bodies
LBD: Lewy body disease
LNs: Lewy neurites
NACC: National Alzheimer’s Coordinating Center
NHWD: non-Hispanic White decedents
PDD: Parkinson disease dementia
SVD: small vessel disease
