# Supplementary figures and images for: The neuropathological landscape of small vessel disease and Lewy pathology in a cohort of Hispanic and non-Hispanic White decedents with Alzheimer disease
Source: Acta Neuropathol Commun. 2024 May 24;12:81. doi: 10.1186/s40478-024-01773-4 (PMC11127432; doi:10.1186/s40478-024-01773-4)

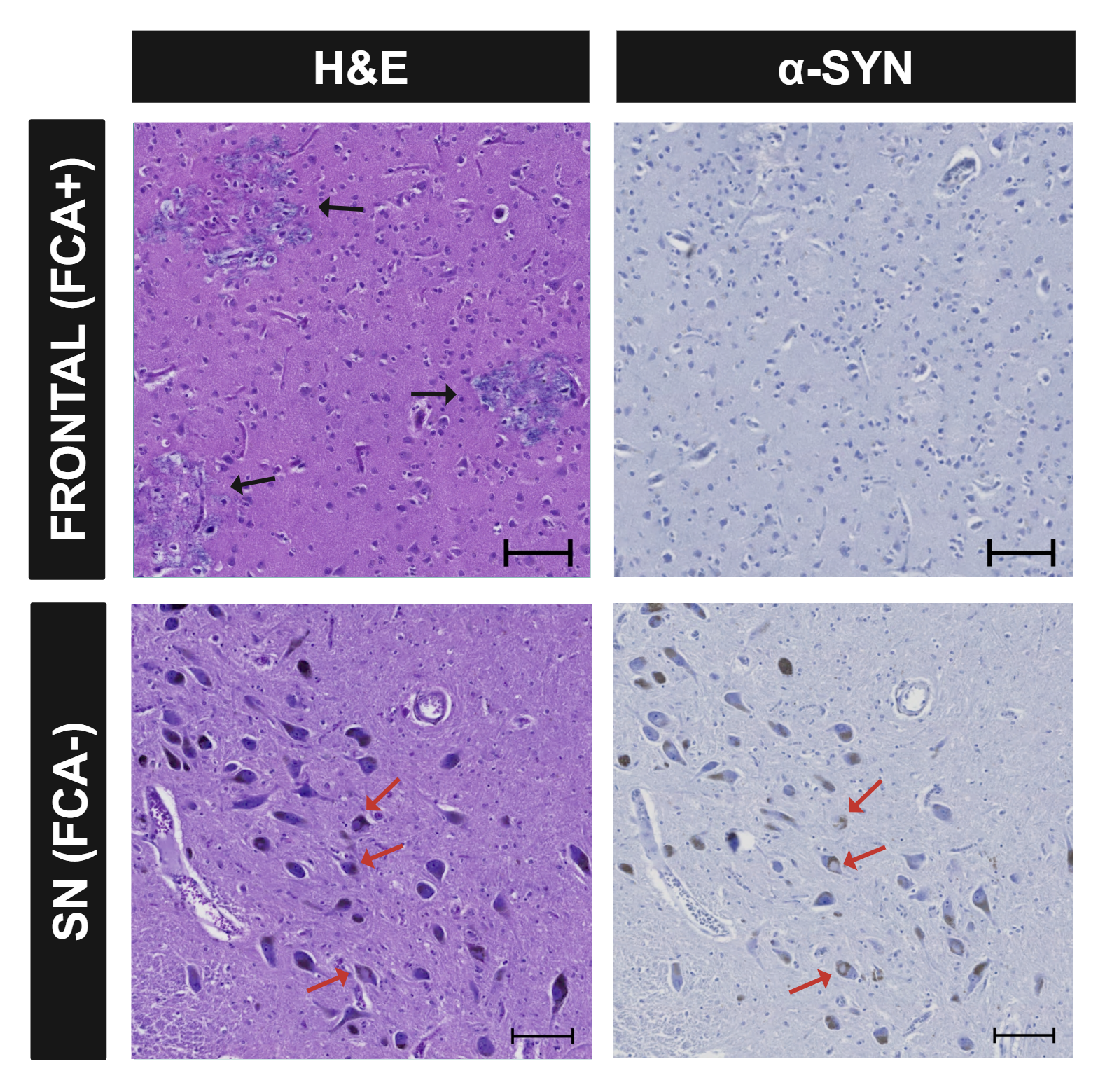

Supplement: Supplementary file 1 — Supplementary Material 1 [file 40478_2024_1773_MOESM1_ESM.png]
